# Evidence for Significant Skew and Low Heritability of Competitive Male Mating Success in the Yellow Fever Mosquito *Aedes aegypti*

**DOI:** 10.1111/eva.70061

**Published:** 2024-12-26

**Authors:** Claudia A. S. Wyer, Vladimir Trajanovikj, Brian Hollis, Alongkot Ponlawat, Lauren J. Cator

**Affiliations:** ^1^ Department of Life Sciences Imperial College London Ascot UK; ^2^ Department of Biological Sciences University of South Carolina Columbia South Carolina USA; ^3^ Department of Entomology Armed Forces Research Institute of Medical Sciences Bangkok Thailand

**Keywords:** *Aedes aegypti*, heritability, mating, scramble competition, sexual selection

## Abstract

*Aedes aegpyti* mosquitoes are vectors of several viruses of major public health importance, and many new control strategies target mating behaviour. Mating in this species occurs in swarms characterised by male scramble competition and female choice. These mating swarms have a male‐biased operational sex ratio, which is expected to generate intense competition among males for mating opportunities. However, it is not known what proportion of swarming males successfully mate with females, how many females each male is able to mate with, and to what extent any variation in the male mating success phenotype can be explained by genetic variation. Here, we describe a novel assay to quantify individual male mating success in the presence of operational sex ratios characteristic of *Ae. aegypti*. Our results demonstrate that male mating success is skewed. Most males do not mate despite multiple opportunities, and very few males mate with multiple females. We compared measures of male mating success between fathers and sons and between full siblings to estimate the heritability of the trait in the narrow $\left(h^{2}\right)$ and broad $\left(H^{2}\right)$ sense, respectively. We found significant broad sense heritability estimates but little evidence for additive genetic effects, suggesting a role for dominance or epistatic effects and/or larval rearing environment in male mating success. These findings enhance our understanding of sexual selection in this species and have important implications for mass‐release programmes that rely on the release of competitive males.

## 1 Introduction

*Aedes aegpyti* is the mosquito vector of several viruses of global health importance, such as yellow fever, chikungunya, dengue, Zika and emerging zoonotic diseases such as Rift Valley fever (Mweya et al. 2013). Both the intensity and distribution of these infections are increasing. For example, there are estimated to be around 390 million dengue infections alone every year (Bhatt et al. 2013) and confirmed cases of locally transmitted dengue fever in the United States (Current Year Data 2023) and France (Fournet et al. 2023) are the most recent reminders of continued geographic expansion of these infections. Options for preventing and treating infections in patients are limited. Therefore, control of *Aedes* populations is a key part of disease prevention (Wilson et al. 2020). *Aedes* mosquitoes and the viral pathogens that they transmit are primarily controlled with the use of chemical insecticides. However, resistance of *Aedes* mosquitoes to the main classes of chemical insecticides is widespread (Ranson et al. 2010). The urgent need for novel control strategies has led to the development of promising new mosquito control interventions.

Several of these new mosquito control methods target mating behaviour (Cator, Wyer, and Harrington 2021). *Ae. aegypti* mosquitoes form small aerial swarms in the vicinity of potential blood‐meal hosts (Oliva, Damiens, and Benedict 2014; Hartberg 1971). Mating occurs mid‐flight and lasts just a few seconds (Hartberg 1971; Yuval 2006; Roth 1948). Within swarms, males compete for access to females (Alcock and Thornhill 2014), and it appears females ultimately choose which male to mate with, displaying decisive rejection behaviours such as kicking and abdominal tilts (Aldersley and Cator 2019; Cator and Harrington 2011; Helinski and Harrington 2012). Not only can mating swarms be targeted with traps (Assogba et al. 2022) or novel insecticides (Conway, Haslitt, and Swarts 2023), but the release of laboratory‐reared males can introduce pathogen resistance (to reduce the prevalence of disease in the mosquito population) (e.g., Conway, Haslitt, and Swarts 2023) or sterility (to reduce the abundance of mosquitoes) (e.g., Ross 2021; Zheng et al. 2019) into wild populations. The success of these ‘reproductive control tools’ is critically dependent on the ability of laboratory‐reared males to compete with wild males and successfully mate with wild females (Cator, Wyer, and Harrington 2021).

Despite the importance of understanding both the distribution and determinants of male mating success, studies investigating this remain scarce. Males outnumber females in swarms (Hartberg 1971; Cator et al. 2011) and have the capacity to mate multiple times, while data suggest that females are largely monandrous (Degner and Harrington 2016; Richardson et al. 2015). Thus, the operational sex ratio (OSR) is skewed towards males. Under these conditions, it would be expected that a given male has a low chance of mating and an even lower chance of mating more than once. What little data exist on male mating success support this assumption. Field observations indicate that just 7.2% (± 0.95% SE) of *Anopheles gambiae* males participating in a swarm were able to mate (Diabaté et al. 2011). While it is assumed that the minority of males within a swarm contribute to the majority of matings (Cator, Wyer, and Harrington 2021), this skew has never been empirically demonstrated in *Ae. aegypti*.

The absence of reliable estimates of male mating success can be attributed, at least in part, to the challenges associated with tracking successful mating attempts within the swarm. Instead, variance in male mating success is most commonly inferred from single reproductive traits, such as insemination capacity (League et al. 2021), sperm production and sperm transfer (Ponlawat and Harrington 2007, 2009). However, the relationship between these proxies and male mating success is unclear (Cator, Wyer, and Harrington 2021). Further, in many taxa, male mating success is based on multiple cues (Candolin 2003; Bradbury and Vehrencamp 2011), and making inferences about the benefits of female choice based on individual traits can introduce substantial bias (Prokop and Drobniak 2016). Prokop and Drobniak (2016) argue that ‘true’ attractiveness can only be determined by scoring female responses to potential mates.

As with any phenotypic trait, variation in male mating success arises as a product of both environmental and genetic variation (Lynch and Walsh 1998; Falconer and Mackay 1996). The extent to which variation in a trait is determined by environmental or genetic factors is a central question in evolutionary biology. Several studies have sought to characterise the relationship between environment and male mating success (or proxies thereof) in mosquitoes. Many of these studies have focused on either larval nutrient availability (Cator and Zanti 2016; Lang et al. 2018; Aldersley et al. 2019; Ng'habi et al. 2008) or larval density (Ng'habi et al. 2005). Larval rearing conditions are frequently studied environmental factors due to their impact on life history traits in adult stages (Briegel, Knüsel, and Timmermann 2001; Chambers and Klowden 1990; Yahouédo et al. 2014), with larval diet consistently demonstrated to affect adult size in *Ae. aegypti* (Aldersley et al. 2019; Telang et al. 2012; Yan, Kibech, and Stone 2021; Nasci and Mitchell 1994; MacLeod, Dimopoulos, and Short 2021) and other mosquito species (Takken et al. 2013; Vantaux et al. 2016; Akoh, Aigbodion, and Kumbak 2011).

The role of genetic factors in male mosquito mating success has only recently begun to be characterised. Laboratory colonisation has been shown to reduce genetic variation (Ng'habi et al. 2015; Gloria‐Soria et al. 2019; Norris et al. 2001), negatively impacting both male reproductive physiology (Baeshen et al. 2014) and male mating success (Ross, Endersby‐Harshman, and Hoffmann 2018). There is also evidence that laboratory adaptation can affect mating behaviour and success in *Anopheles* mosquitoes (Ekechukwu et al. 2015). Qureshi et al. (2019) experimentally evolved populations of *Ae. aegypti* in the presence of sexual selection (multiple males competing to mate with a single female) and documented higher relative mating success compared to males evolved in the absence of sexual selection (a single male paired with a single female), likely driven by the pervasive effects of genetic drift (Wyer, Cator, and Hollis 2023). This suggests a heritable genetic basis to mating success in *Ae. aegypti* (Falconer and Mackay 1996).

In this study we aimed to quantify the variance in male mating success from a recently field‐derived strain of *Ae. aegypti* from Thailand (Thai strain). Since polygynous mating systems with male‐biased OSRs are typically characterised by a reproductive skew, we hypothesised that our population of males exhibit a skewed distribution of competitive mating success. We also hypothesised that since male mating success has been shown to evolve rapidly (Ekechukwu et al. 2015), there is a genetic basis to the variation in male mating success, that the mating success of fathers would significantly predict the mating success of their sons, and that males within full‐sib families more closely resemble each other in terms of mating success than males from different families. We developed an assay to track the competitive mating success of individual males over multiple days in a captive population. These experiments utilised transgenic male mosquitoes with fluorescent protein‐tagged sperm to determine male mate identity when examining mated females, allowing free‐flight mating behaviour (Smith et al. 2007). By performing experiments treating overall male mating success as the phenotype of interest and allowing free‐flight females to choose their mates, the data encompass all pre‐insemination targets of sexual selection. We then used this assay to estimate both broad‐sense $\left(H^{2}\right)$ and narrow‐sense $\left(h^{2}\right)$ heritability.

## 2 Materials and Methods

### 2.1 *Aedes aegpyti* Lines and Rearing

**FIGURE 1:**
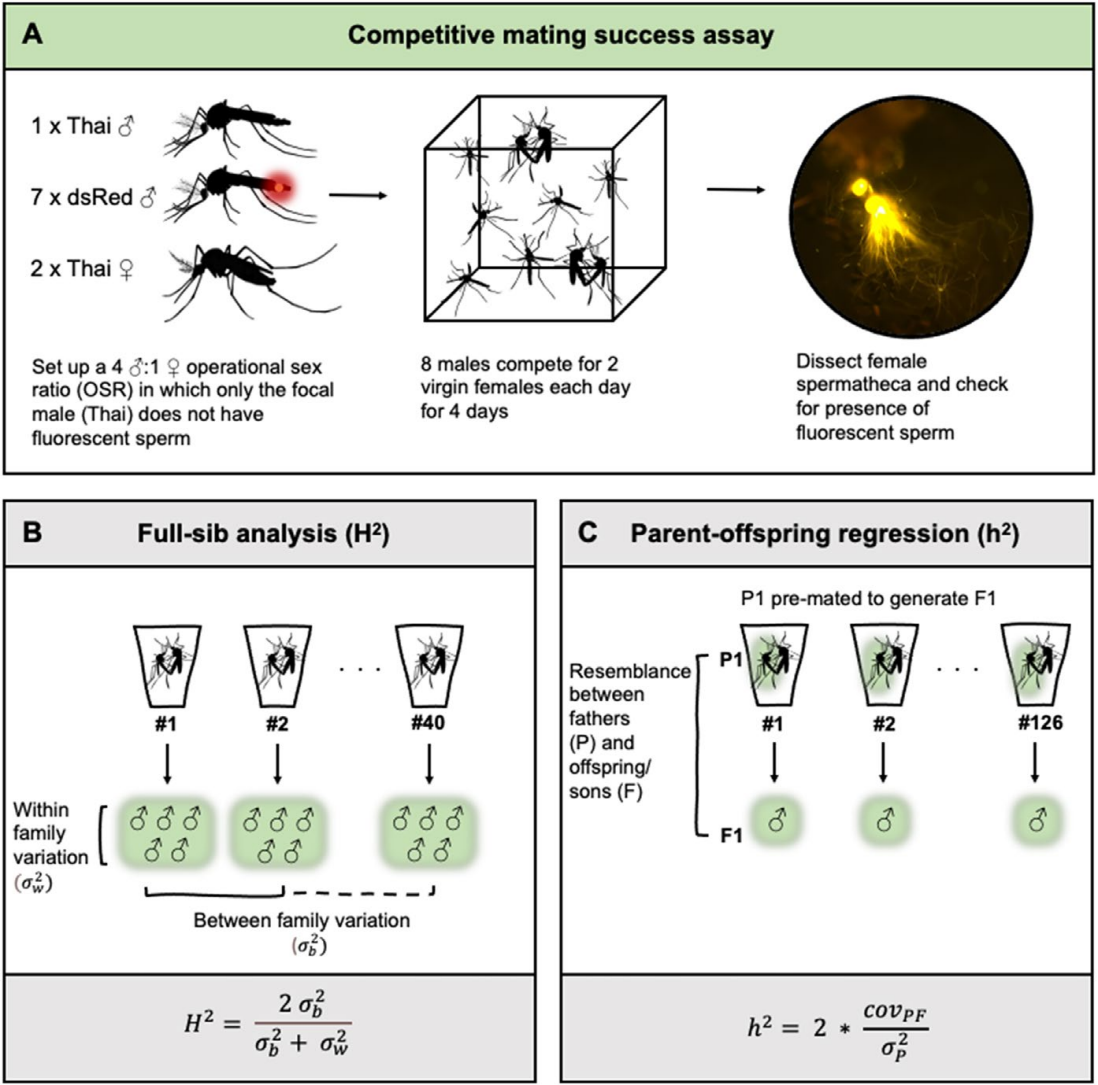
Experimental design. Schematic describing the competitive mating success assay (A), and how it was used for estimating variation and heritability of mating success for full‐sib families of males (B) and parent‐offspring regression of fathers and sons (C). Broad‐sense heritability (*H* ^2^) is calculated in the full‐sib analysis as the between‐family component as a proportion of the total variance. Narrow‐sense heritability (*h* ^2^) is the covariance of offspring and parents, and $\sigma_{P}^{2}$ is the variance of the parents. Since only one male parent is used in each family, the narrow‐sense heritability is twice the slope. In both the full‐sib analysis and parent‐offspring regression, males subjected to the competitive mating success assay are highlighted in green.

In order to determine the identity of the male a female mated with, two genetically distinct *Ae. aegypti* lines were used for this experiment. We quantified variance in the mating success of *a Ae. aegypti* line that was originally collected from Kamphaeng Phet Province, Thailand, by Alongkot Ponlawat in 2021, which underwent just six generations in the lab (F6). As the competitor males, we used a transgenic line of *Ae. aegypti* expressing two transgenes: one that labels sperm with the red fluorescent protein dsRed (Aaβ2t::DsRed) expressed under a testes‐specific promotor, allowing for confirmation of insemination in females mated by males of dsRed genetic background, and another that expresses green fluorescent protein (GFP) under an eye‐specific promotor of larvae of both sexes (3*x*P3::GFP) which serves as an integration marker (Smith et al. 2007) (Figure 1A, Figure S1). The initial dsRed *Ae. aegypti* line was crossed to the Thai *Ae. aegypti*, which was screened for a GFP marker in the eyes of dsRed individuals. The crossing was performed to generate a homozygous dsRed line of *Ae. aegypti* with a similar genetic background to the Thai line. dsRed *Ae. aegypti* (hereafter dsRed) and Thai *Ae. aegypti* eggs were separately vacuum hatched for 20 min. The newly hatched larvae were provided with 0.1 mg of ground fish food overnight (Cichlid Gold [#04328], Hikari, Himeji City, Japan). The following day, L1 larvae were separated into trays of 500 individuals in 2 L of water and provided with fish food *ad libitum*. Once pupated, both strains were sorted by sex and held in separate cages at 27°C, 80% relative humidity on a D12:N12 (with 30 min of dawn/dusk separating each phase) circadian cycle. Adults were provided 10% w/v sucrose *ad libitum*.

### 2.2 Quantification of Male Competitive Mating Success

Male mating success was assessed using an assay design intended to incorporate ecologically relevant aspects of male mating behaviour (Figure 1A), that is, repeated measures of the same individuals and the presence of male competition and female choice. The assay described here was used in both the full‐sib and the half‐sib breeding designs detailed below. At 3 days post‐emergence, seven virgin dsRed males were added to a 19 cm^3^ test cage along with a focal Thai male, provided with 10% w/v sucrose solution and allowed 24 h to acclimate. The following day, the mating competition assay began. The sucrose solution was removed, and two virgin female Thai mosquitoes were aspirated into each cage at 0930 h, allowing 8 h for males to compete for mating opportunities. At 1730 h, the two females were removed and transferred to a 16 oz. holding cup labelled with the same number as the test cage and provided with 10% w/v sucrose solution. Males were provided with 10% w/v sucrose solution and allowed to rest overnight. The following day, the process of adding two virgin females was repeated for a further three days until a total of eight females had been added and removed from each test cage.

The females used in the competitive mating assay were anaesthetised on wet ice and their spermathecae were dissected in 1× phosphate‐buffered saline (PBS) under a stereoscope, and the presence or absence of sperm was recorded using a brightfield microscope. The presence of sperm in the spermathecae confirmed mating status, the presence of fluorescent sperm under a dsRed filter in the spermathecae confirmed insemination by a dsRed (competitor) male and the presence of non‐fluorescent sperm confirmed insemination by the Thai (focal) male. The number of females with non‐fluorescent sperm in the spermathecae (of a possible eight) corresponded to the score of the focal male. This value was also calculated as a proportion of females that were mated over the four days, and hereafter referred to as ‘competitive mating success’.

### 2.3 Broad Sense Heritability

Broad sense heritability ($H^{2}$) describes the extent to which phenotypes are determined by genotypes. A $H^{2}$ value of 0 indicates that none of the variation in the phenotype of the study population is determined by genetics, and a $H^{2}$ value of 1 means that all the variation in the phenotype of the study population is determined by genetics. We performed a modified full‐sib breeding design adapted from a previous study in *Ae. aegypti* (Ware‐Gilmore et al. 2023) to determine the broad sense heritability $\left(H^{2}\right)$ of male mating success in the Thai population of *Ae. aegypti* (Figure 1B). To generate mated females, approximately 75 three‐ to five‐day‐old virgin Thai females were held in a 19 cm^3^ cage with approximately 200 three‐ to five‐day‐old virgin Thai males for 24 h. The following day the females were fed on defibrinated horse blood (FirstLink, UK), and 50 visibly engorged females were aspirated into separate 50 mL centrifuge tubes with the lids modified to hold a cone of damp filter paper, on which the females deposited their eggs. The individual egg clutches were counted, dried and stored. The offspring of those females that had laid > 30 eggs were reared as described previously to pupae, sex sorted and five males from each family kept in 16 oz. holding cups and provided 10% w/v sucrose *ad libitum*. In total 40 families, each consisting of five sons, were assayed using the competitive mating assay described above (and illustrated in Figure 1A) over six experimental blocks.

A nested analysis of variance (ANOVA) was used to estimate $H^{2}$ from the scores (as a proportion of mated females) of five full‐sibs from each of the 40 families. $H^{2}$ was calculated using the variance between ($\sigma_{b}^{2}$) and within $\left(\sigma_{w}^{2}\right)$ full‐sib families as (Falconer and Mackay 1996):

(1) $$H^{2}=\frac{2\;\sigma_{b}^{2}}{\sigma_{b}^{2}+\sigma_{w}^{2}}$$

### 2.4 Narrow Sense Heritability

Heritability in the narrow sense ($h^{2}$) is the extent to which phenotypes are determined by the additive effects of genes transmitted from parents to offspring. We used a parent‐offspring breeding design to estimate $h^{2}$. Two days post‐emergence single virgin focal Thai males were transferred to pre‐mating cages (19 cm^3^) and allowed to mate two virgin Thai females. This pre‐mating step was to confirm that the males were capable of mating and to generate eggs for the F1 generation, which would also be subjected to the competitive mating assay. The following day, females were removed and held in separate 16 oz. cups where they were blood fed and allowed to oviposit on damp filter paper. The eggs were dried and stored as the sons from these clutches would also be assayed. Focal Thai males were then transferred to test cages for the competitive mating success assay described above.

To assess heritability of male mating success as a trait, the competitive mating success assay was repeated for the sons of the focal males (Figure 1C). Eggs laid by one of the randomly selected pre‐mated females were hatched and reared as described previously. Eggs were hatched in separate containers and 10 larvae/container were reared until the pupal stage, when they were separated by sex. A single male offspring per father was subjected to the same pre‐mating to two females and then the same mating competition assay. Females from the mating competition assay were dissected and scores assigned to each son. Competitive mating success of 126 fathers and their sons was measured over four experimental blocks.

These data were used to estimate ($h^{2}$) directly from twice the slope of the parent–offspring regression of 126 father‐son pairs using a linear model and the R package lme4 (Bates et al. 2014).

In addition to estimating $h^{2}$, we explored the effect of mating success of fathers on that of sons as a categorical variable (mated or unmated). The focal male was assigned ‘mated’ if it mated one or more females in competition. The focal male was assigned ‘unmated’ if no females were mated in competition. A chi‐squared test was performed in R (version 4.0.3) using the wald.test function in the aod library (Lesnoff and Lancelot 2008).

### 2.5 Statistical Analysis

All statistical analyses were performed in R (version 4.0.3). Inequality (skew) in mating success was measured using Morisita's non‐standardised index $\left(I_{\delta }\right)$ (Morisita 1962). The Morisita index was proposed by Wade (1995) and Tsuji and Tsuji (1998) to measure monopolisation of reproductive success, and has been cited as the best estimator of the potential for sexual selection (Fairbairn and Wilby 2001). Inequality in mating success was calculated for the P1 and F1 generation of males, and for males from all full‐sib families using the following formula (Morisita 1962; Tsuji and Tsuji 1998):

(2) $$I_{\delta }=N\;\frac{\sum \left(x^{2}\right)-\sum x}{{\left(\sum x\;\right)}^{2}-\sum x}$$

where N is group size, ∑x is the sum of matings over all mating individuals, and $\sum x^{2}$ is the sum of all mating counts squared. $I_{\delta }$ = 1 when the distribution is random, $I_{\delta }$ < 1 based on a uniform distribution, and $I_{\delta }$ > 1 when the distribution is skewed. The maximum possible value of $I_{\delta }$ is equal to N.

Deviation from randomness can be checked by comparing the test statistic with $\chi^{2}$ (df = N−1) (Morisita 1962; Tsuji and Tsuji 1998):

(3) $$\chi^{2}=I_{\delta }\;\left(\sum x-1\;\right)+N-\sum x$$

### 2.6 Ethical Note

No ethical approval was required for the species used in this research.

## 3 Results

### 3.1 Variation in Male Mating Success

**FIGURE 2:**
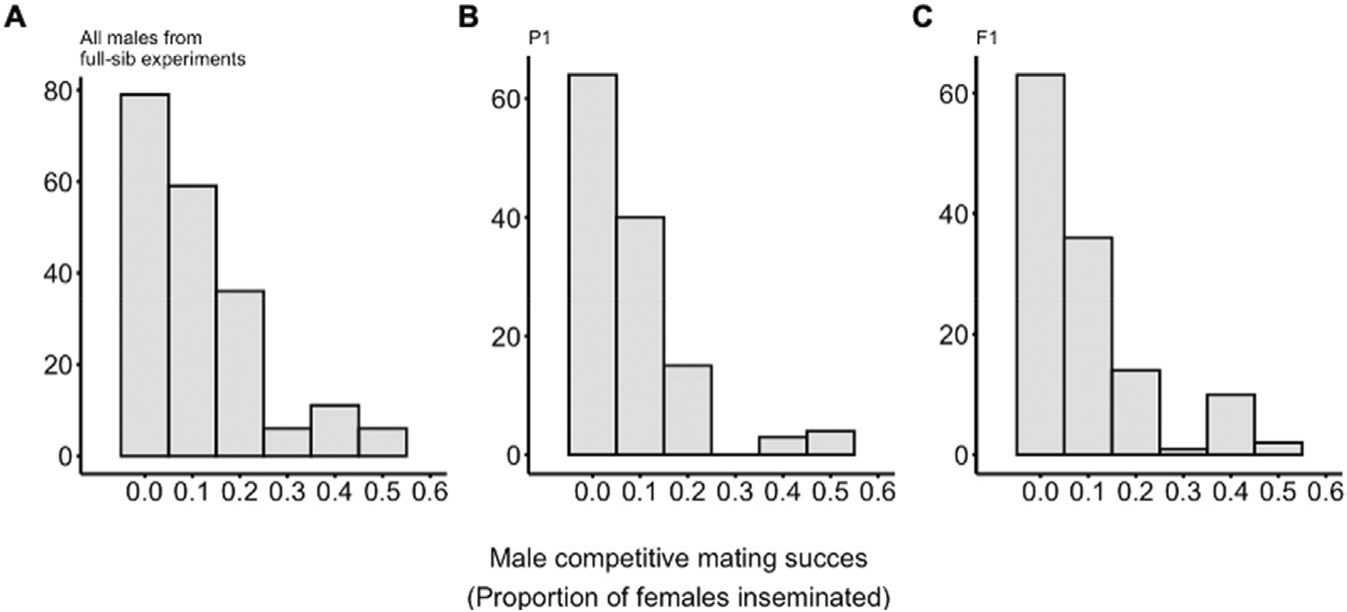
Distribution of competitive mating success for males from parent‐offspring and full‐sib experiments. (A) The distribution of competitive mating success for 200 males from 40 full‐sib families. (B) The distribution of competitive mating success for 126 fathers (P1) randomly selected from a laboratory population of *Ae. aegypti* and (C) their 126 sons (F1). For all experiments, competitive mating success was measured as the proportion of total females mated (of a possible eight) by the focal male.

Across all experiments and generations, we found significant evidence of skew in male mating success. In broad sense heritability experiments, the modal score for all males from 40 full‐sib families was 0; 39.5% of full‐sib males (*n* = 79) did not mate a single female, 33% of full‐sib males (*n* = 66) mated a single female, and 27.5% of full‐sib males (*n* = 55) mated multiple females (Figure 2A). On average, males mated with approximately one female (mean = 1.035, SD = 1.127). The Morisita index indicated a positive and significant skew in mating success ($I_{\delta }$ = 1.219, *p* < 0.05).

Similarly, in the narrow sense assays, the distribution of male mating success as a proportion of inseminated females was right‐skewed, with most males not mating with a single female, and very few mating multiple times (Figure 2B,C). The distribution of competitive mating success was similar for fathers and for sons (two‐sample Kolmogorov–Smirnov test: *D* = 0.048, *p* = 0.999). Though males had access to up to eight virgin females, the range of the proportion of females mated was 0–0.5 for both fathers and sons. Overall, the most common outcome (the modal score) was 0; 46.83% of fathers (*n* = 59) and 50% of sons (*n* = 63) did not mate in competition. 34.13% of fathers (*n* = 43) and 28.57% of sons (*n* = 36) mated a single female, and only 19.05% of fathers (*n* = 24) and 21.43% of sons (*n* = 27) mated multiple females. On average, focal males mated with less than one female (fathers: mean = 0.817, SD = 0.991; sons: mean = 0.825, SD = 1.028). Under the male‐biased sex ratio of this mating assay, the Morisita index for mating scores of fathers indicated a positive but non‐significant skew in mating success ($I_{\delta }$ = 1.247, *p* > 0.05). The data collected on sons exhibited a positive and significant skew in mating success ($I_{\delta }$ = 1.341, *p* < 0.05).

### 3.2 Heritability of Male Mating Success

**FIGURE 3:**
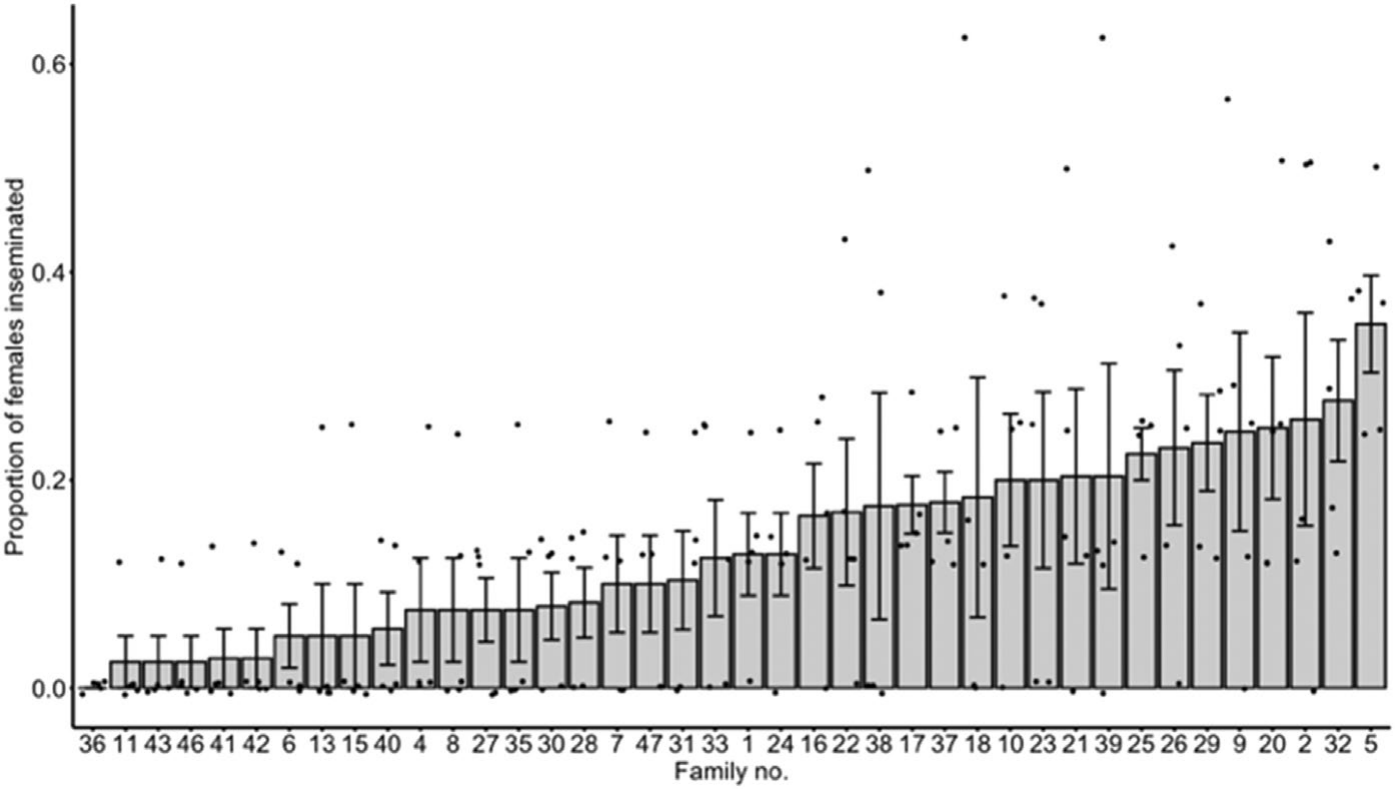
Variation in male competitive mating success. Mean ± SEM proportion of virgin females inseminated by focal males of *n* = 40 *Ae. aegypti* families. Black circles represent the proportion of females inseminated by individual males (*n* = 5) within each family.

From the full‐sib analysis, we generated an estimate of broad‐sense heritability ($H^{2}$) of 0.694, suggesting that 69.4% of the variance in competitive mating success is due to genetic differences among males. A one‐way ANOVA revealed a statistically significant difference in the proportion of females mated between at least two families (*F* (39, 160) = 2.1808, *p* < 0.001; Figure 3). We also observed a statistically significant difference in male mating success between the best and worst performing families, that is, those with the highest and lowest mean mating success, respectively (two‐sample *t*‐test between family #5 and family #36: *t* = 7.4833, df = 8, *p* < 0.001), and also between the 10% of families from the extreme ends of the distribution of male mating success (two‐sample *t*‐test between top 10% and bottom 10% of families *t* = 11.21, df = 6, *p* < 0.001).

**FIGURE 4:**
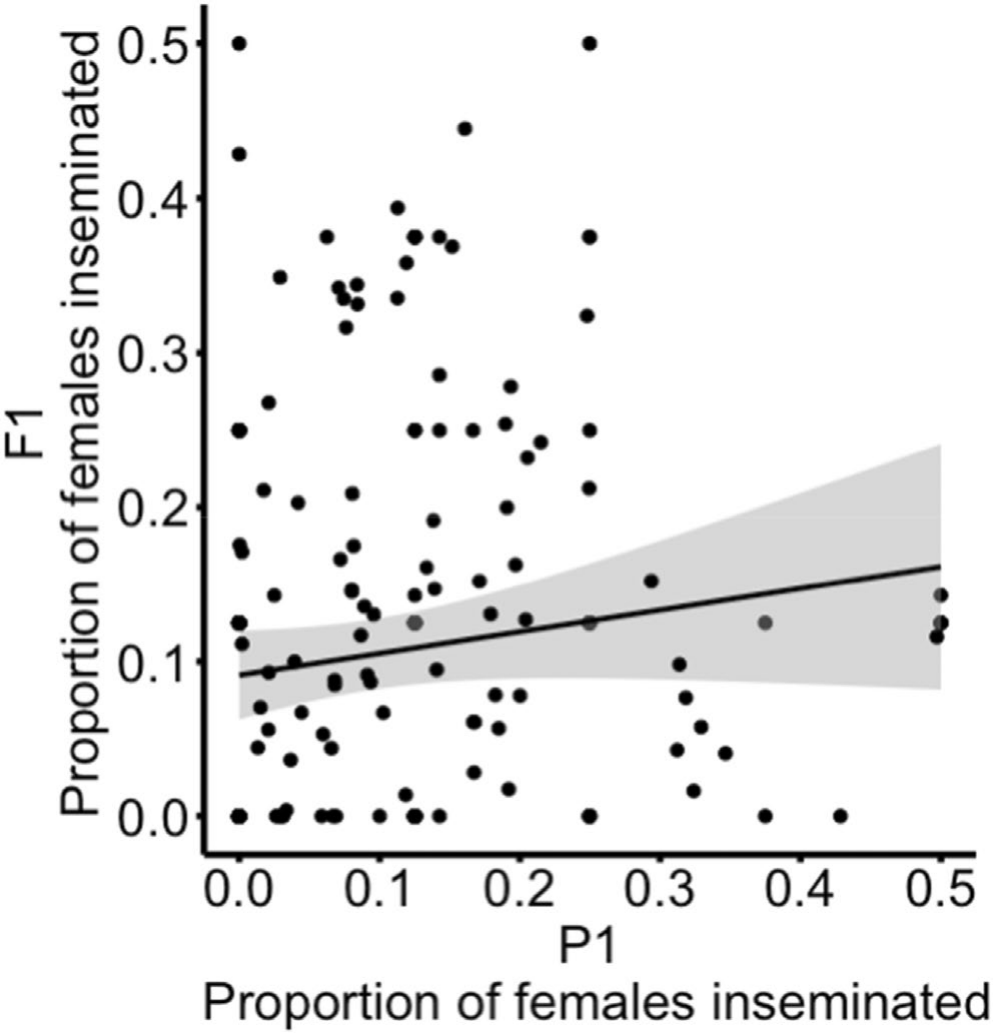
Father‐son regression analysis of competitive mating success. Relationship between the proportion of matings achieved by fathers and the proportion of matings achieved by sons (*R* ^2^ = 0.017; *p* = 0.141). The solid line is the linear regression of competitive mating success between fathers and sons with standard error in grey. Points have been jittered to prevent overlapping using the ggplot function geom_jitter.

In the analysis of competitive mating success for 126 father‐son pairs, we observed a positive ($h^{2}$ = 0.2, 95% CI [−0.042, 0.289]), but not significant relationship between the mating success of fathers and their sons (Figure 4; lm: *R* ^2^ = 0.017; *p* = 0.141). Even when the outcome of the competitive mating success assay was converted to a binary categorical variable (i.e., where males were either ‘mated’ or ‘unmated’), the overall effect of the fathers' mating success on the sons' mating success was not significant (Table 1: *χ* ^2^ = 0.8, df = 1, *p* = 0.37).

**TABLE 1:** Two‐way contingency table of fathers and sons mating statuses.

	P1 mating status	
	Mated	Unmated
F1 mating status		
Mated	36	27
Unmated	31	32

## 4 Discussion

Understanding male mating success is critical to the effective deployment of reproductive control strategies. Here, we use an assay with ecologically relevant levels and mechanisms of sexual selection to characterise the skew and heritability of male mating success for a recently colonised laboratory population of *Ae. aegypti*.

We empirically demonstrate the degree of skew affecting a population of *Ae. aegypti* experiencing sexual selection in the form of female choice and mate competition. Under a 4:1 male‐biased OSR, males from the P1, F1 and full‐sib families all showed positive skew indexes ($I_{\delta }$ = 1.247, $I_{\delta }$ = 1.341 and $I_{\delta }$ = 1.219, respectively) indicating an unequal partitioning of competitive mating success among males in the population. Our data indicate that in serial competition, not only do a small proportion of males achieve multiple matings, but many males do not mate a single female, despite multiple mating opportunities.

The distribution and presence of significant skew in male mating success have two important implications for reproductive control strategies. First, it suggests that insemination capacity, a commonly used metric to quantify male mating success (Gwadz and Craig 1968; Jones and Pilitt 1973; Foster and Lea 1975; Boyer et al. 2011), may not be a relevant proxy of indicator quality in mass‐rearing facilities. While males have been shown to be able to inseminate between 3.5 (± 0.07) and 11.5 (± 0.53) females in the absence of competition (reviewed in Cator, Wyer, and Harrington 2021), Our results indicate that in competitive scenarios, most males do not mate a single female, and mating multiple females is rare.

Second, our measures suggest that competitive male mating success is not a lottery. Most males will fail and a smaller minority than expected by chance will be successful. Thus, it is critical that research into what factors determine male mating success continue.

We investigated the role of genetic factors in determining male mating success as a phenotypic trait. The broad sense heritability estimate of male competitive mating success in the full‐sib experimental design was relatively high ($H^{2}$ = 0.694), which indicates that most of the variation among individuals in this population is due to genetic factors. Other studies in insects have reported similarly high $H^{2}$ estimates for traits related to mating. For example, heritability of the propensity to store apyrene sperm, the duration of the mating refractory period, and the degree of polyandry were all found to be high in the green‐veined white butterfly, *Pieris napi* ($H^{2}$ = 0.734, 0.846 and 0.627, respectively) (Wedell 2001; Wedell, Wiklund, and Cook 2002). This is the first time that resemblance of mating success among full‐sib relatives has been measured in *Ae. aegypti*, though several other studies have identified $H^{2}$ for other traits in mosquitoes, such as thermal stress response ($H^{2}$ = 0.14) (Ware‐Gilmore et al. 2023), and susceptibility to dengue ($H^{2}$ = 0.4) and chikungunya ($H^{2}$ = 0.18) viruses (Novelo et al. 2023).

Caution must be taken when interpreting $H^{2}$ estimates as they are subject to significant potential for bias. For instance, full‐sib males are raised in a common environment, which may increase their phenotypic similarity beyond genetic effects, inflating heritability ($H^{2}$) estimates. Future studies could rear males separately to reduce this shared environment effect. Additionally, maternal environmental effects are a potential source of increased resemblance between full‐sibs (Falconer and Mackay 1996). In *Ae. aegypti*, maternal larval nutrition has been shown to affect both lipid investment in offspring (Yanchula and Alto 2021) and offspring development time (Zirbel and Alto 2018). Though all mosquito larvae were reared with food provided *ad libitum*, it is possible that other maternal effects may have influenced the phenotypic values of related males.

We used a father‐son regression to estimate narrow‐sense heritability and found a low estimate of competitive mating success heritability ($h^{2}$ = 0.2), which was not significantly different from zero. Narrow‐sense heritability is an indicator of an important source of genetic variation, additive genetic variance. Additive genetic variance is the component of the genetic variation captured by the $H^{2}$ estimate that is responsible for resemblance between parents and offspring, and it is the basis for the response to selection (Falconer and Mackay 1996).

It is perhaps not unexpected that we did not find significant $h^{2}$ for male mating success. This result is consistent with the low heritability of behaviour in *Drosophila* (0.18 ± 0.03 SE) (Roff and Mousseau 1987) and across multiple taxa (0.235; 95% CI: 0.200–0.271) (Dochtermann et al. 2019). It is also consistent with the low narrow‐sense heritability means calculated in a meta‐analysis of multi‐component ‘suite’ of sexually selected traits (0.12 ± 0.05 SE), such as attractiveness (Prokuda and Roff 2014). Though the targets of sexual selection in *Ae. aegypti* are unknown, there is evidence to suggest that several types of traits, across multiple sensory modalities might be involved (Adams et al. 2021; Wang et al. 2021; Andrés et al. 2020; Callahan, Ross, and Hoffmann 2018), as is the case for many taxa (Candolin 2003).

It may be in this species that other types of non‐additive genetic variance are more important for total genetic variation. The high $H^{2}$ estimate we derived from the full‐sib experiment would include dominance variance and interaction variance. Dominance variance increases the resemblance between full‐sibs relative to the resemblance between parents and offspring, which is also likely to exaggerate heritability estimates (Falconer and Mackay 1996). The difficulty in parsing out the additive genetic variation from other sources of variation mean that $H^{2}$ should be considered an upper‐limit of heritability (Falconer and Mackay 1996).

It may also be that there was significant narrow sense heritability, and we had an insufficient number of father‐son pairs in the assay to detect it. Sampling error of heritability estimates is inversely proportional to the relationship of individuals squared, the number of families measured and the number of individuals within a family (Falconer and Mackay 1996). As such, thousands of observations are often required to generate precise heritability ($h^{2}$) estimates. Whilst this assay has clear advantages in terms of ecological relevance and precision in determining the competitive mating success of individual males, it is not a high‐throughput approach and so problematic for generating enough trait values for generating reliable estimates of heritability.

We argue that the lower estimate of heritability $\left(h^{2}\right)$ derived from the parent‐offspring regression is likely more reliable than the estimate of heritability for the full‐sib families ($H^{2}$). Firstly, trait covariance between fathers and sons is not inflated by a shared environment, given that measuring competitive mating success at a given age necessitates that they are assayed separately; a more variable environment reduces heritability. Secondly, offspring do not share dominance variation with their parents, as is the case for full sibs, meaning that resemblance between fathers and sons is generated solely by additive genetic variation. Other studies have identified similarly large differences between heritability estimates derived from full‐sib families and from parent‐offspring regressions. For example, in a study of the sheepshead minnow, *Cyprinodon variegatus* , estimated heritability of resistance to chemical environmental contaminants was very high when obtained from a full‐sib analysis (mean $H^{2}$ = 0.846), whereas a parent‐offspring regression yielded very low heritability estimates (mean $h^{2}$ = 0.091) (Klerks and Moreau 2001). The authors of this study attributed this striking disparity in heritability values to substantial dominance and common environmental effects (Klerks and Moreau 2001).

Despite apparently, though perhaps unsurprisingly, low heritability ($h^{2}$) identified by the parent‐offspring regression, previous work has shown that male mating success has the potential to evolve. Qureshi et al. (2019) showed that under intense sexual selection male mating success in *Ae. aegypti* could evolve in just five generations of experimental evolution, indicating additive genetic variation in male mating success. Whilst it is plausible that this study design was not sufficiently sensitive to detect additive genetic variation in a single generation, there is also a substantial body of evidence to suggest that heritability ($h^{2}$), the fraction of phenotypic variance that is additive, is not an accurate measure of evolutionary potential (Hansen, Pélabon, and Houle 2011; Houle 1992). Hansen, Pélabon, and Houle (2011) demonstrated that the inherent positive correlation between additive genetic variance and other components of phenotypic variance (e.g., environmental or dominance variance) may cancel out the relationship between additive genetic variance and heritability, and may explain why the correlation between evolvability and heritability is essentially zero. Additionally, a low $h^{2}$ estimate does not necessarily indicate a low level of genetic determination, and it may simply be that of all the observed variation, additive genetic variation constitutes a small proportion of it.

## 5 Conclusions

Polygynous mating systems, where males can mate multiple times and females generally mate once, are expected to exhibit a skewed mating success. While the aerial mating swarms of *Ae. aegypti* have been characterised as polygynous (Cator, Wyer, and Harrington 2021), variance in mating success has previously only been estimated using proxies of this trait, often in the absence of sexual selection. This study employed an assay designed to estimate the variation and heritability of male mating success, which encompassed active female mate choice and male–male competition. The study provides new evidence for reproductive skew, and for the potential for sexual selection in this species. Indeed, most males fail to mate in competition, and only a small fraction of males successfully mate with multiple females. This result holds significant implications for the reproductive control of *Ae. aegypti*, as the success of such control strategies depends on the mating success of released laboratory‐reared males. Additionally, this study indicated a high upper limit for heritability ($H^{2}$) of male mating success, though a small (non‐significant) proportion of the phenotypic variance is attributed to additive genetic variation, in line with expectations of the heritability of multivariate traits. Future studies should focus on identifying the specific traits underlying male mating success, and the relative contribution of environment to competitive male mating success.

## Conflicts of Interest

The authors declare no conflicts of interest.

## Supporting information


Appendix S1.

## Data Availability

Data are available as Supporting Information.
