# Supplementary material for: Evidence for Significant Skew and Low Heritability of Competitive Male Mating Success in the Yellow Fever Mosquito Aedes aegypti
Source: Evol Appl. 2024 Dec 26;17(12):e70061. doi: 10.1111/eva.70061 (PMC11671345; doi:10.1111/eva.70061)
Supplement: Supplementary file 1 — Appendix S1. [file EVA-17-e70061-s001.zip › Evolutionary_applications_supporting_figures.docx]

**

**Figure S1. *Ae. aegypti* dsRed phenotype.** (A) Dissected male testes and accessory glands (x 10 magnification). (B) Dissected spermathecae of an inseminated female. One spermatheca is ruptured and individual sperm are visible (x 10 magnification).
